# Whole-Genome Sequencing of *Pseudoalteromonas* sp. Strain KAN5, an Agar-Degrading Bacterium Isolated from the Gut of an Alga-Eating Fish

**DOI:** 10.1128/mra.00398-22

**Published:** 2022-07-07

**Authors:** Yuka Kawase, Takuma Tanabe, Makoto Kawamukai, Masa-aki Yoshida

**Affiliations:** a Oki Marine Biological Station, Faculty of Life and Environmental Sciences, Shimane University, Oki, Shimane, Japan; b Faculty of Life and Environmental Sciences, Shimane University, Matsue, Shimane, Japan; Montana State University

## Abstract

A strain of *Pseudoalteromonas* that degrades agar was isolated from the intestines of an alga-eating fish (Andamia tetradactyla). We named the strain KAN5 and report on the genome sequenced with the Oxford Nanopore Technologies platform. The 3.8-Mbp genome contains 3,428 protein-coding genes, and the genes involved in agar degradation were confirmed.

## ANNOUNCEMENT

The genus *Pseudoalteromonas* comprises Gram-negative marine bacteria (1). Pseudoalteromonas atlantica is one of the bacteria that acts as a primary producer of biofilms and is known to have an *agrA* gene, encoding an extracellular β-agarase (2). In this study, we report the complete genome sequence (3,899,889 bp; GC content, 41.0%) of *Pseudoalteromonas* sp. strain KAN5, which was isolated from the gut of the alga-eating fish Andamia tetradactyla (3).

Single colonies were collected from the glycerol stock of the isolate and incubated overnight in marine broth 2216 (Becton, France) at 37°C. Genomic DNA was extracted using the NucleoSpin microbial DNA kit (TakaraBio, Japan) according to the manufacturer's protocol. After isolation, the 16S rRNA V4 region was amplified to identify the species using the 515f/808r primer set (4) and was found to be similar to that of the genus *Pseudoaleromonas*. For accurate identification, molecular phylogenetic analysis based on the full 16S rRNA sequence retrieved from the complete genome revealed that the most similar strain was *Pseudoalteromonas* sp. strain MMM18 (GenBank accession number AY187028.1) (99.22% identity, according to NCBI BLASTn [June 2022]). Default parameters were used for all software unless otherwise noted.

To determine the whole-genome sequence of *Pseudoalteromonas* sp. strain KAN5, a sequencing library was prepared using the SOK-RAD004 rapid sequencing kit (Oxford Nanopore Technologies [ONT], UK) with transposase-based fragmentation. Long-read sequencing was performed using a MinION flow cell. The quality of the sequences was checked using the MinKNOW interface v20.06.4 (ONT). Guppy v4.5.3 was used to base call the sequences. A total of 5,068,244 kbp were obtained, with an average read length of 6,385 bp, corresponding to approximately 1,300-fold coverage of the entire KAN5 genome. We performed quality filtering for assembly and subsampled the reads in order of length using Filtlong v0.2.1 (https://github.com/rrwick/Filtlong). Reads with a length of more than 20,000 bases were subsampled, and a total of 1 Gbp were used for assembly (with the following Filtlong options: –target_bases 1,000,000,000; –min_length 20,000). The reads were assembled into a single linear contig using Canu v1.9 (5). Using Racon v1.4.20 (6), we polished assembly errors four times for all reads. Annotation was performed using DDBJ DFAST (7). The total genome length was 3,899,889 bp, and the GC content was 41.0%. A total of 3,428 protein-coding sequences, 101 tRNA genes, and 28 rRNA genes were predicted. The strain of *Pseudoalteromonas* closest to KAN5 was Pseudoalteromonas shioyasakiensis strain SDCH90 (GenBank accession number NZ_CP077770.1), based on *dnaA* sequence similarity (85.0% identity at the nucleotide level, according to NCBI BLASTn [April 2022]). The average nucleotide identity (ANI) calculator was used to compare the two prokaryotic genome sequences (8) and found that the overall OrthoANIu value was 76.11%.

We verified the β-agarase genes in the genome. One agarase candidate was 99% identical to *Pseudoalteromonas agrA* (GenBank accession number KPZ59655.1) at the protein level (according to NCBI BLASTp [April 2022]). Another glucosyl hydrolase locus, encoding glycoside hydrolase 16 (GH16) of the GH family, was shown to match the sequence of S1727 in *Pseudoalteromonas* (GenBank accession number WP_138554011.1) (100% identity at the protein level, according to NCBI BLASTp [December 2021]). In addition, there are two genes near GH16 in the genome that are presumed to be GH1 and GH3. The N-terminal side of GH3 has a signal peptide attached to it, suggesting that it is secreted extracellularly. These genes are presumably related to the ability of KAN5 to degrade seaweed-derived polysaccharides.

### Data availability.

The assembled bacterial chromosome draft sequence was deposited in DDBJ under the accession number AP025589. Raw ONT reads are available in the DDBJ Sequence Read Archive (DRA) under DRA accession number DRR345756. Sample information is also available under BioProject accession number PRJDB12987.
